# Sex-Specific Differences in End-of-Life Burdensome Interventions and Antibiotic Therapy in Nursing Home Residents With Advanced Dementia

**DOI:** 10.1001/jamanetworkopen.2019.9557

**Published:** 2019-08-16

**Authors:** Nathan M. Stall, Hadas D. Fischer, Kinwah Fung, Vasily Giannakeas, Susan E. Bronskill, Peter C. Austin, Jeremy N. Matlow, Kieran L. Quinn, Susan L. Mitchell, Chaim M. Bell, Paula A. Rochon

**Affiliations:** 1 ICES, Toronto, Ontario, Canada; 2 Women's College Research Institute, Women's College Hospital, Toronto, Ontario, Canada; 3 Institute of Health Policy, Management and Evaluation, University of Toronto, Toronto, Ontario, Canada; 4 Department of Medicine, University of Toronto, Toronto, Ontario, Canada; 5 Division of General Internal Medicine and Geriatrics, Sinai Health System, Toronto, Ontario, Canada; 6 Hebrew SeniorLife Institute for Aging Research, Boston, Massachusetts

## Abstract

**Importance:**

Nursing home residents with advanced dementia have limited life expectancies yet are commonly subjected to burdensome interventions at the very end of life. Whether sex-specific differences in the receipt of these interventions exist and what levels of physical restraints and antibiotics are used in this terminal setting are unknown.

**Objective:**

To evaluate the population-based frequency, factors, and sex differences in burdensome interventions and antibiotic therapy among nursing home residents with advanced dementia.

**Design, Setting, and Participants:**

This population-based cohort study from Ontario, Canada, used linked administrative databases held at ICES, including the Continuing Care Resident Reporting System Long-Term Care database, which contains data from the Resident Assessment Instrument Minimum Data Set, version 2.0. Nursing home residents (n = 27 243) with advanced dementia who died between June 1, 2010, and March 31, 2015, at 66 years or older were included in the analysis. Initial statistical analysis was completed in May 2017, and analytical revisions were conducted from November 2018 to January 2019.

**Exposure:**

Sex of the nursing home resident.

**Main Outcomes and Measures:**

Burdensome interventions (transitions of care, invasive procedures, and physical restraints) and antibiotic therapy in the last 30 days of life.

**Results:**

The final cohort included 27 243 nursing home residents with advanced dementia (19 363 [71.1%] women) who died between June 1, 2010, and March 31, 2015, at the median (interquartile range) age of 88 (83-92) years. In the last 30 days of life, burdensome interventions were common, especially among men: 5940 (21.8%) residents were hospitalized (3661 women [18.9%] vs 2279 men [28.9%]; *P* < .001), 2433 (8.9%) had an emergency department visit (1579 women [8.2%] vs 854 men [10.8%]; *P* < .001), and 3701 (13.6%) died in an acute care facility (2276 women [11.8%] vs 1425 men [18.1%]; *P* < .001). Invasive procedures were also common; 2673 residents (9.8%) were attended for life-threatening critical care (1672 women [8.6%] vs 1001 men [12.7%]; *P* < .001), and 210 (0.8%) received mechanical ventilation (113 women [0.6%] vs 97 men [1.2%]; *P* < .001). Among the 9844 residents (36.1%) who had a Resident Assessment Instrument Minimum Data Set, version 2.0, completed in the last 30 days of life, 2842 (28.9%) were physically restrained (2002 women [28.3%] vs 840 men [30.4%]; *P* = .005). More than one-third (9873 [36.2%]) of all residents received an antibiotic (6599 women [34.1%] vs 3264 men [41.4%]; *P* < .001). In multivariable models, men were more likely to have a transition of care (adjusted odds ratio, 1.41; 95% CI, 1.33-1.49; *P* < .001) and receive antibiotics (adjusted odds ratio, 1.33; 95% CI, 1.26-1.41; *P* < .001). Only 3309 residents (12.1%; 2382 women [12.3%] vs 927 men [11.8%]) saw a palliative care physician in the year before death, but those who did experienced greater than 50% lower odds of an end-of-life transition of care (adjusted odds ratio, 0.48; 95% CI, 0.43-0.54); *P* < .001) and greater than 25% lower odds of receiving antibiotics (adjusted odds ratio, 0.74; 95% CI, 0.68-0.81; *P* < .001).

**Conclusions and Relevance:**

In this study, many nursing home residents with advanced dementia, especially men, received burdensome interventions and antibiotics in their final days of life. These findings appear to emphasize the need for sex-specific analysis in dementia research as well as the expansion of palliative care and end-of-life antimicrobial stewardship in nursing homes.

## Key Points

**Question:**

What are the population-based frequency, factors, and sex-specific differences in burdensome interventions and antibiotic therapy at the very end of life among nursing home residents with advanced dementia?

**Findings:**

In this population-based cohort study of 27 243 decedent nursing home residents with advanced dementia, men were statistically significantly more likely than women to experience burdensome interventions (ie, transitions of care, invasive procedures, and physical restraints) and receive antibiotics; only a minority of residents saw a palliative care physician in the year before death, but those who did were significantly less likely to experience an end-of-life transition of care and to receive antibiotics.

**Meaning:**

Study findings underscore the importance of sex-specific analysis in dementia research and the expanding roles of palliative care and antimicrobial stewardship in the nursing home setting.

## Teaser

This population-based cohort study examines the sex-based variations in burdensome interventions (care transitions, invasive procedures, and use of physical restraints) and antibiotic therapy at the very end of life among nursing home residents with advanced dementia in Ontario, Canada.

## Introduction

In Canada, approximately 564 000 people were living with dementia in 2016, a number that is expected to increase to 937 000 by 2031.^1^ In addition, most persons with dementia are older adults (≥65 years), two-thirds of whom are women.^1^ In the advanced stages of the disease, nursing homes become a common site of care and death.^2,3,4,5^

Persons with advanced dementia typically have profound memory impairment, minimal verbal communication, loss of ambulatory abilities, an inability to perform activities of daily living, and urinary and fecal incontinence.^3,6^ Nursing home residents with advanced dementia have a median survival of 1.3 years, yet they are commonly subjected to burdensome interventions toward the end of life.^7^ Burdensome interventions include a variety of transitions between health care facilities, invasive procedures, and physical restraints. These interventions are often avoidable, may not improve comfort, and are frequently distressing to residents and their families.^3,8,9,10,11,12^

Research from the United States demonstrates high rates of burdensome interventions among nursing home residents with advanced dementia, including transitions of care,^10,11,12^ medications of questionable benefit,^13,14^ invasive procedures,^8^ and parenteral therapy and tube feeding.^7^ Although this body of work has improved understanding of the terminal management of advanced dementia, it has not integrated sex-specific analyses despite known sex differences in the prevalence, risk factors, and clinical presentation of dementia as well as calls from major research institutes and academic journals to explore sex differences in the field of dementia research.^15,16,17^ In addition, the population-level use of physical restraints and antibiotics in this terminal setting has not been described, raising concerns about the frequency of potentially inappropriate use at the very end of life.^18^ Antibiotic therapy is potentially burdensome in the setting of advanced dementia because the intravenous or intramuscular route of administration can be associated with discomfort and pain, and the therapy itself is associated with increased risk of drug-drug interactions, antibiotic-associated diarrhea, and *Clostridium difficile* infection that could lead to preventable prescribing cascades.^19,20^

In light of these knowledge gaps, we conducted a population-based cohort study to describe the frequency and sex-specific differences in burdensome interventions (ie, transitions of care, invasive procedures, and physical restraints) and potentially burdensome antibiotic therapy among decedent nursing home residents with advanced dementia.

## Methods

### Study Design 

This population-based cohort study of decedent nursing home residents with advanced dementia was conducted in Ontario, Canada. Ethics approval was obtained from the Research Ethics Board of Sunnybrook Health Sciences Centre, Ontario, Canada. Informed consent was not required in accordance with Section 45 of Ontario’s Personal Health Information Protection Act. This study followed the Strengthening the Reporting of Observational Studies in Epidemiology (STROBE) reporting guideline and the Reporting of Studies Conducted Using Observational Routinely-Collected Health Data (RECORD) statement guidelines.^21,22^

### Data Sources

The study used linked administrative databases held at ICES, including the Continuing Care Resident Reporting System Long-Term Care database. This database contains mandatory clinical assessment information on all nursing home residents, and the data are collected with the validated Resident Assessment Instrument Minimum Data Set, version 2.0 (RAI-MDS 2.0).^23,24^ The RAI-MDS 2.0 is completed on admission to a nursing home, quarterly, and after any substantial health change.^25^ We also used other provincial databases to capture resident demographics, prescription medication claims, physician services, and hospital and emergency department (ED) use (eAppendix 1 in the Supplement). These data sets were linked using unique encoded identifiers and analyzed at ICES and have been used extensively in previous research on transitions of care and antibiotic therapy among older individuals in nursing homes.^26,27,28,29^ Data-sharing agreements prohibit ICES from making the data set publicly available, but access to the data set may be granted to those who meet prespecified criteria for confidential access. The full data set creation plan and underlying analytic code are available from the authors. Section 45 of Ontario’s Personal Health Information Protection Act authorizes ICES to collect personal health information without patient consent for the purpose of analyzing or compiling statistical information to manage, evaluate or monitor, allocate resources to, or plan all or part of the Canadian health system.

### Derivation of Study Cohort

We identified a cohort of all nursing home residents with advanced dementia who died between June 1, 2010, and March 31, 2015, and who were 66 years or older on the date of death. We derived a decedent cohort rather than following older adults from nursing home admission to death given that the clinical course of advanced dementia in this setting has been previously described.^7^ In the present study, we focused on burdensome interventions and antibiotic therapy at the very end of life, which has not been comprehensively described at the population level.^7^ Residents were eligible for inclusion if they had at least 1 RAI-MDS 2.0 assessment completed in the 30 to 120 days before death to ensure they received a comprehensive assessment before death (Figure); if residents had more than 1 assessment, the most recent or closest to death was selected. Age of at least 66 years on the date of death was required to ensure a full year of prior medication use was available for the look-back. We required that all residents in the cohort lived in their nursing home for at least 30 days before death.

**Figure.**
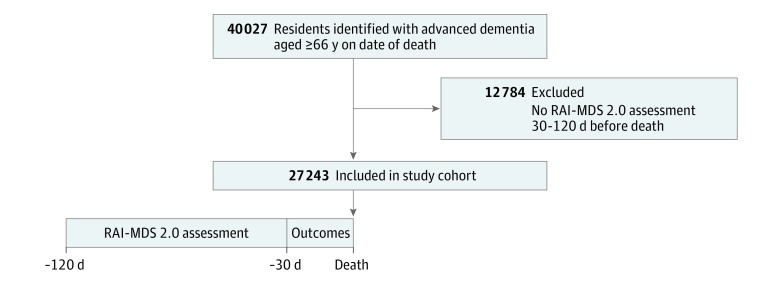
Participant Flow and Outcome Assessment All included residents had at least 1 completed Resident Assessment Instrument Minimum Data Set, version 2.0 (RAI-MDS 2.0) within 30 to 120 days before their death. Burdensome interventions and antimicrobial use were measured in the last 30 days of a resident’s life.

History of dementia was obtained from either (1) the documentation of dementia on any RAI-MDS 2.0 assessment any time during the 5 years before death, (2) any physician diagnostic codes associated with an outpatient or inpatient visit for dementia within the 5 years before death, or (3) any prescription of a cholinesterase inhibitor within the year before death.^30,31,32^ Advanced dementia was identified as a Cognitive Performance Scale score of 5 or higher on the last available RAI-MDS 2.0 assessment between 30 and 120 days before death.^33^

### Primary Exposure and Outcomes

The primary exposure was sex of the nursing home resident. The health administrative databases at ICES capture sex (biological attributes) rather than gender (socially constructed roles, behaviors, expressions, and identities associated with each sex).^34^

The primary outcomes were burdensome interventions (transitions of care, invasive procedures, and physical restraints) and potentially burdensome antibiotic therapy. Selection of these interventions was based on a review of the literature and the expert opinions of geriatric, internal, and palliative medicine specialists.^3,7,35^ The antibiotic outcome was labeled separately as potentially burdensome because guidance on the use of antimicrobials in dementia at the end of life has only recently emerged.^19,36^

A transition of care was defined as any ED visit or acute care hospitalization.^37^ Invasive procedures included mechanical ventilation, medical attendance for life-threatening critical care, and intravenous hydration therapy. Physical restraints were defined using the RAI-MDS 2.0 quality indicators, which included the use of at least 1 trunk restraint, limb restraint, or chair designed to prevent rising.^38^ Because the intravenous hydration and physical restraints outcomes were measured in the RAI-MDS 2.0 during the 7 days before the assessment, we reported these outcomes only for residents who had an RAI-MDS 2.0 assessment completed in the last 30 days of life. Antibiotics included all systemic antibiotics administered via the enteral or parenteral route and excluded antifungals, antivirals, and topical agents.^39,40^ The full details of the Ontario health administrative data sources are included in eAppendix 1 in the Supplement.

### Nursing Home and Resident Characteristics

We measured nursing home size (number of beds stratified into 3 groups: 1-99, 100-199, and ≥200) and location (urban vs rural) because these facility characteristics are known to be associated with transitions of care and antibiotic therapy.^29,41^ A detailed set of resident-level characteristics were ascertained from the Continuing Care Resident Reporting System Long-Term Care database as well as hospital, ED, and physician claims databases.^28,42,43^ Time between admission to nursing home and death was calculated for each individual.

### Statistical Analysis

Summary statistics and univariate analyses were computed to compare, by resident sex, baseline nursing home resident characteristics, end-of-life burdensome interventions, and potentially burdensome antibiotic use. We created separate multivariable statistical models for transitions of care, attendance for life-threatening critical care, physical restraints, and antibiotic therapy. All models were adjusted for the same nursing home–level (urban vs rural location and number of beds) and resident-level (age, activities of daily living score, bedbound status, low-income status, geriatric medicine or palliative care assessment in the year before death, and malignancy in the preceding 5 years) factors, which were selected a priori on the basis of a review of the literature and the expert opinions of geriatric, internal, and palliative medicine specialists.^3,7,35^

Multivariable generalized estimating equations were used to measure the association between sex and burdensome interventions and antibiotic therapy to account for potential differences between nursing homes and the clustering of residents within them. Initial statistical analysis was completed in May 2017, with analytical revisions performed from November 2018 to January 2019. All analyses were performed using SAS statistical software, version 9.4 (SAS Institute Inc). Tests were 2-tailed, and the level of statistical significance was set at α = .05.

## Results

The study included 27 243 nursing home residents with advanced dementia (with a median [interquartile range] age at death of 88 [83-92] years, and with 19 363 women [71.1%] and 7880 [28.9%] men) who died between June 1, 2010, and March 31, 2015 (Table 1). At the time of death, women were more likely than men to be older (median [interquartile range] age, 89 [84-93] years vs 85 [80-89] years; *P* < .001) and to have lived for a longer time in the nursing home (median [interquartile range] time, 4.2 [2.2-6.7] years vs 2.7 [1.3-4.8] years; *P* < .001). Most residents were dependent or totally dependent for their activities of daily living (15 595 women [80.5%] vs 5822 men [73.9%]), and 2213 [8.1%] were totally bedbound (1659 women [8.6%] vs 554 men [7.0%]). A minority of residents received either palliative care (2382 women [12.3%] vs 927 men [11.8%]) or geriatric medicine (892 women [4.6%] vs 582 men [7.4%]) specialist care in the year before death. Most residents lived in an urban nursing home (16 615 women [85.8%] vs 6695 men [85.0%]) that contained more than 100 beds (15 100 women [78.0%] vs 6182 men [78.5%]).

**Table 1.** Resident-Level and Facility-Level Characteristics

Variable	Total	Women	Men
Resident characteristics			
No. (%)	27 243	19 363 (71.1)	7880 (28.9)
Age at death, median (IQR), y	88 (83-92)	89 (84-93)	85 (80-89)
Charlson Comorbidity Index, median (IQR)	1 (1-3)	1 (1-3)	2 (1-3)
Time between admission to nursing home and death, median (IQR), y	3.7 (1.9-6.2)	4.2 (2.2-6.7)	2.7 (1.3-4.8)
Low-income status, No. (%)	8198 (30.1)	6670 (34.5)	1528 (19.4)
ADL hierarchy scale score, No. (%)			
0-2 (No or limited assistance)	195 (0.7)	122 (0.6)	73 (0.9)
3 (Extensive)	1909 (7.0)	1179 (6.1)	730 (9.3)
4 (Maximal)	3722 (13.7)	2467 (12.7)	1255 (15.9)
5 (Dependent)	8676 (31.8)	6244 (32.3)	2432 (30.9)
6 (Total dependence)	12 741 (46.8)	9351 (48.3)	3390 (43.0)
Bedbound, No. (%)	2213 (8.1)	1659 (8.6)	554 (7.0)
Specialty care assessment in 1 y before death, No. (%)			
Palliative care	3309 (12.1)	2382 (12.3)	927 (11.8)
Geriatric medicine	1474 (5.4)	892 (4.6)	582 (7.4)
Malignancy in the past 5 y, No. (%)	935 (3.4)	538 (2.8)	397 (5.0)
Facility characteristics			
Location, No. (%)			
Urban	23 310 (85.6)	16 615 (85.8)	6695 (85.0)
Rural	3930 (14.4)	2745 (14.2)	1185 (15.0)
No. of beds, No. (%)			
1-99	5961 (21.9)	4263 (22.0)	1698 (21.6)
100-199	14 465 (53.1)	10 356 (53.5)	4109 (52.1)
≥200	6817 (25.0)	4744 (24.5)	2073 (26.3)

Abbreviations: ADL, activities of daily living; IQR, interquartile range.

### Burdensome Interventions

#### End-of-Life Transitions of Care

In the last 30 days of life, 2433 residents (8.9%) had an ED visit (1579 women [8.2%] vs 854 men [10.8%]; *P* < .001), 5940 (21.8%) were hospitalized (3661 women [18.9%] vs 2279 men [28.9%]; *P* < .001), and 3701 (13.6%) died in an acute care facility (2276 women [11.8%] vs 1425 men [18.1%]; *P* < .001) (Table 2). Of the 5980 residents admitted to a hospital, 1498 (25.1%) were hospitalized in the last 3 days of life (928 of 3661 women [25.3%] vs 570 of 2279 men [25.0%]), and 344 (5.8%) had an intensive care unit admission (185 women [5.1%] vs 159 men [7.0%]). The diagnoses most responsible for these hospitalizations included nonspecific medical care, pneumonitis, sepsis, pneumonia, and urinary tract disorders (eAppendix 3 in the Supplement).

**Table 2.** Burdensome Interventions and Antibiotic Therapy During Last 30 Days of Life

Variable	No. (%)			*P* Value
	Total (N = 27 243)	Women (n = 19 363)	Men (n = 7880)	
Burdensome intervention				
Transitions of care				
ED visit	2433 (8.9)	1579 (8.2)	854 (10.8)	<.001
Hospitalization	5940 (21.8)	3661 (18.9)	2279 (28.9)	<.001
Newly hospitalized in last 3 d of life	1498 (5.5)	928 (4.8)	570 (7.2)	<.001
ICU admission	344 (1.3)	185 (1.0)	159 (2.0)	<.001
Place of death				<.001
Nursing home	23 542 (86.4)	17 087 (88.2)	6455 (81.9)	
Acute care facility	3701 (13.6)	2276 (11.8)	1425 (18.1)	
Invasive procedures				
Mechanical ventilation	210 (0.8)	113 (0.6)	97 (1.2)	<.001
Medical attendance for life-threatening critical care	2673 (9.8)	1672 (8.6)	1001 (12.7)	<.001
Intravenous hydration^a^	513 (5.2)	343 (4.8)	170 (6.2)	.002
Physical restraints				
Trunk/limb or chair restraint^a^	2842 (28.9)	2002 (28.3)	840 (30.4)	.005
Potentially burdensome intervention				
Antibiotic therapy prescription				
Any	9873 (36.2)	6599 (34.1)	3264 (41.4)	<.001
1	5434 (19.9)	3641 (18.8)	1793 (22.8)	<.001
2	2605 (9.6)	1726 (8.9)	879 (11.2)	<.001
≥3	1834 (6.7)	1242 (6.4)	592 (7.5)	.001

Abbreviations: ED, emergency department; ICU, intensive care unit.

^a^ Calculated for the 9844 residents (36.1%; 7085 women [36.6%] and 2759 men [35.0%]) in the cohort who had a Resident Assessment Instrument Minimum Data Set, version 2.0, assessment completed in the last 30 days of life.

In multivariable analysis, male sex was associated with 41% higher odds of experiencing at least 1 end-of-life transition of care (composite of ED visits and hospitalizations) after adjusting for all of the other measured facility- and resident-level characteristics (adjusted odds ratio [aOR], 1.41; 95% CI, 1.33-1.49; *P* < .001) (Table 3). Several other factors were statistically significantly associated with end-of-life transitions of care, including seeing a palliative care physician in the year before death (aOR, 0.48; 95% CI, 0.43-0.54; *P* < .001) (Table 3).

**Table 3.** Multivariable Logistic Regression Analysis of Resident-Level and Facility-Level Characteristics Associated With Transitions of Care (Composite of Emergency Department Visits and Acute Care Hospitalizations)

Variable	aOR (95% CI)^a^	*P* Value
Exposure		
Male vs female	1.41 (1.33-1.49)	<.001
Factor		
ADL score 5-6 vs 0-4	0.58 (0.54-0.62)	<.001
Time in nursing home >1 y vs ≤1 y	0.34 (0.29-0.40)	<.001
Low-income status	1.31 (1.23-1.40)	<.001
Geriatric medicine physician assessment in year before death	1.48 (1.31-1.67)	<.001
Malignancy in the preceding 5 y before death	1.05 (0.92-1.21)	.47
Age, per 10 y	0.70 (0.67-0.73)	<.001
Bedbound status	0.68 (0.60-0.76)	<.001
Urban vs rural nursing home location	1.53 (1.30-1.80)	<.001
No. of nursing home beds		
<100	1 [Reference]	
100-199	1.73 (1.28-2.33)	<.001
≥200	2.27 (1.44-3.56)	<.001
Palliative care physician assessment in the year before death	0.48 (0.43-0.54)	<.001

Abbreviations: ADL, activities of daily living; aOR, adjusted odds ratio.

^a^ The multivariable logistic regression analysis modeled the exposure and each factor of interest, adjusting for the exposure and all of the remaining factors.

#### End-of-Life Invasive Procedures

Many residents experienced invasive procedures at the end of life, with 2673 (9.8%) receiving medical attendance for life-threatening critical care (1672 women [8.6%] vs 1001 men [12.7%]; *P* < .001) and 210 (0.8%) receiving mechanical ventilation (113 women [0.6%] vs 97 men [1.2%]; *P* < .001). In multivariable analysis, male sex was associated with 33% higher odds of attendance for life-threatening critical care (aOR, 1.33; 95% CI, 1.22-1.46; *P* < .001) (eAppendix 2 in the Supplement). Among the 9844 residents (36.1%; 7085 women [36.6%] and 2759 men [35.0%]) who had an RAI-MDS 2.0 assessment completed in the last 30 days of life, 513 (5.2%) received intravenous hydration (343 women [4.8%] vs 170 men [6.2%]; *P* = .002) (Table 2).

#### End-of-Life Physical Restraints

Among the 9844 residents (36.1%; 7085 women [36.6%] and 2759 men [35.0%]) who had an RAI-MDS 2.0 assessment completed in the last 30 days of life, many were physically restrained, with 2842 (28.9%) being placed in either a trunk restraint, limb restraint, or chair designed to prevent rising (2002 women [28.3%] vs 840 men [30.4%]; *P* = .005) (Table 2). In multivariable analysis, male sex was associated with 17% higher odds of being physically restrained (aOR, 1.17; 95% CI, 1.07-1.31; *P* < .001) (eAppendix 2 in the Supplement).

### Potentially Burdensome Interventions: End-of-Life Antibiotic Therapy

In the last 30 days of life, 9873 residents (36.2%; 6599 women [34.1%] vs 3264 men [41.4%]; *P* < .001) received at least 1 antibiotic; 5434 (19.9%; 3641 women [18.8%] vs 1793 men [22.8%]; *P* < .001) received 1 antibiotic prescription, 2605 (9.6%; 1726 women [8.9%] vs 879 men [11.2%]; *P* < .001) received 2 unique antibiotic prescriptions, and 1834 (6.7%; 1242 women [6.4%] vs 592 men [7.5%]; *P* = .001) received 3 or more unique antibiotic prescriptions (Table 2).

In multivariable analysis, male sex was associated with 33% higher odds of receiving any antibiotic after adjusting for all of the other measured facility- and resident-level characteristics (aOR, 1.33; 95% CI, 1.26-1.41; *P* < .001) (Table 4). Several other factors were statistically significantly associated with receipt of antibiotics, including seeing a palliative care physician in the year before death, which was associated with 26% lower odds of receiving an antibiotic (aOR, 0.74; 95% CI, 0.68-0.81; *P* < .001) (Table 4).

**Table 4.** Multivariable Logistic Regression Analysis of Resident-Level and Facility-Level Characteristics Associated With Any Antibiotic Therapy

Variable	aOR (95% CI)^a^	*P* Value
Exposure		
Male vs female	1.33 (1.26-1.41)	<.001
Factor		
ADL score 5-6 vs 0-4	0.97 (0.91-1.04)	.40
Time in nursing home >1 y vs ≤1 y	0.75 (0.65-0.87)	<.001
Low-income status	1.08 (1.02-1.14)	.009
Geriatric medicine physician assessment in the year before death	1.10 (0.97-1.24)	.13
Malignancy in the preceding 5 y before death	0.86 (0.75-0.99)	.03
Age, per 10 y	0.95 (0.92-0.98)	.004
Bedbound status	0.87 (0.79-0.96)	.004
Urban vs rural nursing home location	1.12 (1.01-1.25)	.03
No. of nursing home beds		
<100	1 [Reference]	
100-199	0.88 (0.73-1.06)	.19
≥200	0.83 (0.62-1.10)	.19
Palliative care physician assessment in the year before death	0.74 (0.68-0.81)	<.001

Abbreviations: ADL, activities of daily living; aOR, adjusted odds ratio.

^a^ The multivariable logistic regression analysis modeled the exposure and each factor of interest, adjusting for the exposure and all the remaining factors.

## Discussion

This study of nearly 30 000 decedent nursing home residents with advanced dementia confirms substantial rates and sex-specific differences in burdensome interventions and potentially burdensome antibiotic therapy at the end of life. In the last 30 days of life, nearly 1 in 10 residents visited an ED, more than 1 in 5 were hospitalized, and 1 in 7 died in an acute care setting. In addition, almost 1 in 10 residents received medical attendance for life-threatening critical care; more than 1 in 4 were physically restrained; and more than 1 in 3 received antibiotics, with several individuals receiving multiple unique antibiotic prescriptions. Men were statistically significantly more likely than women to experience all burdensome interventions and receive antibiotics. Only a minority of residents saw a palliative care physician in the year before death, but those who did were significantly less likely to experience an end-of-life transition of care and to receive antibiotics.

These sex-specific findings are important and provide a more comprehensive perspective on the management of end-of-life nursing home residents with advanced dementia. The results of this Canadian study are in line with those of previous studies in US nursing homes, which demonstrate comparable rates of end-of-life burdensome procedures and transitions of care.^7,12^ This work extends other findings by reporting on physical restraint use, which is increasingly recognized as a burdensome intervention at the end of life.^35^ In addition, although 2 previous small cohort studies reported high rates of antibiotic prescription among nursing home residents with advanced dementia at the end of life, the present study confirms these rates in a large population-based cohort.^18,44^

A particular strength of this study is the sex-specific analysis, which demonstrates substantial differences between women and men in the receipt of end-of-life burdensome interventions and antibiotics. We found that, compared with women, men in this cohort had much higher rates of burdensome interventions and antibiotic therapy and were significantly more likely to die in an acute care facility.

The sex-specific findings are consistent with those of a systematic review of differences in hospitalization of nursing home residents, which reported that men have consistently higher rates of hospitalization.^45^ These findings merit particular attention and future study, especially because the reasons for these variations are poorly understood.^46^ The limited research on sex-based differences in the clinical presentation of dementia reports that men have more aggressive behaviors, more comorbidity, and higher mortality compared with women.^47,48,49^ These factors may have implications for the provision of potentially burdensome interventions and antibiotics, but the sex-specific differences observed remained consistent even after adjustment.^43,50^

Therefore, we hypothesized that the observed differences may be more associated with gender (sociocultural factors) than with sex (biological factors).^34^ First, women are more likely than men to engage in advanced care planning and discuss their wishes with family and friends.^51^ Women also have less fear of death and more commonly refuse intubation and cardiopulmonary resuscitation.^52^ Second, women are more likely to assume caregiving roles for a spouse with dementia; the stressful and demanding nature of the caregiving relationship may complicate end-of-life decision-making and motivate more intensive care at the very end of life.^53,54,55^ Outside of the caregiving relationship, men may be more likely to have women advocating for their care than vice versa and may therefore receive more aggressive care. Third, gender-based inequities in the receipt of physical and mental health care among persons with dementia have been well described, with men being more likely to receive primary and consultative care.^56^ Fourth, research in other areas highlights how physicians’ implicit gender bias is a factor in women being less likely than men to be offered invasive procedures.^57,58^ Findings of the present study suggest that this gender bias may persist at the end of life, when more aggressive care—something that may have been valued at a younger age—is still provided despite such care no longer being optimal.

The widespread use of potentially burdensome end-of-life antibiotic therapy also merits a discussion. In the setting of advanced dementia, whether antibiotics confer any life-prolonging or symptomatic advantage is unclear, and they are often prescribed in the absence of strong evidence for bacterial infection despite the established Minimum Criteria for the Initiation of Antibiotics in Residents of Long-term Care Facilities.^7,59,60,61^ Most proxies of nursing home residents with advanced dementia are also unaware of and do not participate in the decision-making for management of suspected infections.^62^ The indiscriminate use of antibiotics in nursing homes is associated with high rates of multidrug-resistant organism colonization and is a major public health threat given that residents are frequently hospitalized.^44^ Our study findings and these considerations highlight the expanding role for antimicrobial stewardship in the nursing home setting and in persons with advanced dementia.^19^

The study results also underscore the importance of access to palliative care for nursing home residents with advanced dementia. Only 12.1% of residents in the cohort saw a palliative care physician in the year before death, but those who did so experienced more than 50% lower odds of an end-of-life transition of care and a more than 25% lower odds of receiving antibiotics. These findings were consistent with results in other settings, in which only a minority of residents with advanced dementia accessed palliative care, yet those who did had fewer unmet needs at the end of life.^63,64^

### Limitations 

This study has limitations owing to the use of administrative databases. Although misclassification of antibiotic therapy was unlikely given the accuracy of the database,^40^ we could verify prescription dispensation only and not medication adherence. Misclassification of physical restraint use and burdensome interventions in the nursing home setting was also unlikely, as the RAI-MDS 2.0 database is well validated.^24^ However, our reliance on the RAI-MDS 2.0 database resulted in the exclusion of 12 784 residents who did not have an assessment in the 30 to 120 days before death. Given the lack of information on inpatient medication and physical restraint use in the hospital data sets, we likely underestimated the burden of antibiotic therapy and physical restraints at the end of life.

Another limitation is that the study data precluded us from ascertaining whether end-of-life burdensome interventions and antibiotic therapy were discordant or concordant with residents’ pre-expressed wishes and advanced care planning. Previous work has highlighted, however, that more than 90% of substitute decision-makers for nursing home residents with advanced dementia believed comfort to be the primary goal of care.^7^ Other work has reported similar findings, with most physicians and family members caring for individuals with dementia strongly favoring palliative care for advanced dementia.^60^ In this study, most residents were transferred to the hospital for reasons that would not promote a goal of comfort; the exception was hip fractures, which were uncommon (eAppendix 3 in the Supplement). We acknowledge, however, that what one substitute decision-maker considers burdensome may be deemed appropriate by another substitute decision-maker faced with the same circumstances.

## Conclusions

This population-based cohort study in Ontario, Canada, found that many older nursing home residents with advanced dementia, especially men, received burdensome interventions and potentially burdensome antibiotic therapy at the very end of life. These findings appear to underscore the importance of sex-specific analysis in dementia research and the expansion of palliative care and antimicrobial stewardship in the nursing home setting.

## Notes

Supplement. **eAppendix 1.** Ontario Health Administrative Data Sources Included in This Study **eAppendix 2.** Multivariable Logistic Regression Analysis Evaluating Resident– and Nursing Home–Level Characteristics Associated With Medical Attendance for Life-Threatening Critical Care and Receipt of Physical Restraints in the Last 30 Days of Life **eAppendix 3.** Most Responsible Diagnoses for First Hospitalization in Last 30 Days of Life
