# Supplementary material for: Sex-Specific Differences in End-of-Life Burdensome Interventions and Antibiotic Therapy in Nursing Home Residents With Advanced Dementia
Source: JAMA Netw Open. 2019 Aug 16;2(8):e199557. doi: 10.1001/jamanetworkopen.2019.9557 (PMC6704739; doi:10.1001/jamanetworkopen.2019.9557)
Supplement: Supplement. — eAppendix 1. Ontario Health Administrative Data Sources Included in This Study eAppendix 2. Multivariable Logistic Regression Analysis Evaluating Resident– and Nursing Home–Level Characteristics Associated With Medical Attendance for Life-Threatening Critical Care and Receipt of Physical Restraints in the Last 30 Days of Life eAppendix 3. Most Responsible Diagnoses for First Hospitalization in Last 30 Days of Life [file jamanetwopen-2-e199557-s001.pdf]

Supplementary Online Content

Stall NM, Fischer HD, Fung K, et al. Sex-specific differences in end-of-life burdensome interventions and antibiotic therapy in nursing home residents with advanced dementia. *JAMA Netw Open*. 2019;2(8):e199557. doi:10.1001/jamanetworkopen.2019.9557

eAppendix 1. Ontario Health Administrative Data Sources Included in This Study

eAppendix 2. Multivariable Logistic Regression Analysis Evaluating Resident– and Nursing Home–Level Characteristics Associated with Medical Attendance for Life-Threatening Critical Care and Receipt of Physical Restraints in the Last 30 Days of Life

eAppendix 3. Most Responsible Diagnoses for First Hospitalization in Last 30 Days of Life

This supplementary material has been provided by the authors to give readers additional information about their work.

eAppendix 1. Ontario Health Administrative Data Sources Included in This Study

Ontario provides all residents of nursing homes with universal access to medically necessary physician services, ambulatory and hospital care, and prescription medications under a publicly funded provincial health insurance program.

Database	Description
Continuing Care Reporting System Long-Term Care (CCRS-LTC) database	The CCRS-LTC database is comprised of all mandatory, clinical assessments performed on nursing home residents in Ontario. Nursing home assessments are made using the Resident Assessment Instrument Minimum Data Set (RAI-MDS) version 2.0, a previously validated tool.[1,2]
Discharge Abstract Database (DAD)	The DAD is compiled by the Canadian Institute for Health Information and contains administrative, clinical (diagnoses and procedures), and demographic information for all admissions to acute care hospitals in Ontario.<br><br>DAD records have been demonstrated to have excellent agreement (over 99%) for demographic and administrative data. Regarding diagnoses, median agreement between original DAD records and re-abstracted records for the 50 most common most responsible diagnoses was noted to be 81% (Sensitivity 82%; Specificity 82%).[3] The corresponding median agreement for the 50 most frequently performed surgical procedures was 92% (sensitivity 95%, positive predictive value 91%).
National Ambulatory Care Reporting System (NACRS)	The NACRS is compiled by the Canadian Institute for Health Information and contains administrative, clinical (diagnoses and procedures), and demographic information for all patient visits made to hospital- and community-based ambulatory care centres (emergency departments, day surgery units, dialysis and cancer care clinics) in Ontario.
Ontario Drug Benefit (ODB) program database	The ODB database contains prescription medication claims for those covered under the provincial drug program, mainly those aged 65 years and older, nursing home residents, and those receiving social assistance. Each medication claim has an associated prescriber identifier which indicates the health practitioner who wrote the prescription. A special flag in the ODB database indicates whether the prescription was dispensed in the community or nursing home setting.<br><br>An audit of 5,155 randomly selected prescriptions dispensed from 50 Ontario pharmacies determined that the ODB had an error rate of 0.7% and none of the pharmacy characteristics examined (locations, owner affiliation, productivity) were associated with coding errors.[4]
Ontario Health Insurance Plan (OHIP) physician billing claims database	The OHIP physician billing claims database contains information on all outpatient services provided by fee-for-service physicians in Ontario and "shadow billings" for physicians paid under alternate payment plans. Billing codes are specific in identifying services provided in the nursing home setting.
Registered Persons Database (RPDB)	The RPDB provides basic demographic information (age, sex, area of residence, date of birth, and date of death for deceased individuals) about anyone who has ever received an Ontario health card number (e.g., been enrolled in the province's publicly funded health insurance system).
Ontario Cancer Registry (OCR)	The OCR database contains information on all Ontario residents who have been newly diagnosed with cancer or who have died of cancer. All new cases of cancer are registered, except non-melanoma skin cancer.
ICES Physician Database (IPDB)	The IPDB contains yearly information about all physicians in Ontario including demographics, specialization and workload. The IPDB comprises information from the OHIP Corporate Provider Database (CPDB), the Ontario Physician Human Resource Data Centre (OPHRDC) database and the OHIP database of physician billings.

(1) Kim H, Jung YI, Sung M, Lee JY, Yoon JY, Yoon JL. Reliability of the interRAI Long Term Care Facilities (LTCF) and interRAI Home Care (HC). *Geriatr Gerontol Int*. 2015; 15(2):220-228.
(2) Mor V. A comprehensive clinical assessment tool to inform policy and practice: applications of the minimum data set. *Med Care*. 2004; 42(4 Suppl):III50-III59.
(3) Juurlink D, Preyra C, Croxford R, Chong A, Austin P, Tu J et al. Canadian Institute for Health Information Discharge Abstract Database: A Validation Study. 2006. Toronto, Institute for Clinical Evaluative Sciences.
Ref Type: Report
(4) Levy AR, O'Brien BJ, Sellors C, Grootendorst P, Willison D. Coding accuracy of administrative drug claims in the Ontario Drug Benefit database. *Can J Clin Pharmacol*. 2003; 10(2):67-71.

eAppendix 2. Multivariable Logistic Regression Analysis Evaluating Resident– and Nursing Home–Level Characteristics Associated With Medical Attendance for Life-Threatening Critical Care and Receipt of Physical Restraints in the Last 30 Days of Life

**Exposure**	**Medical attendance for life-threatening critical care aOR (95% CI)[*]**	**Physical restraints aOR (95% CI)[*†]**
Male vs. Female sex	1.33 (1.22–1.46)	1.17 (1.07–1.31)
**Factor**		
Activities of Daily Living score 5-6 vs. 0-4	0.82 (0.75–0.91)	1.96 (1.72–2.24)
Time in nursing home >1 year vs. ≤1 year	0.61 (0.48–0.77)	1.71 (1.27–2.31)
Low income status	1.26 (1.15–1.39)	0.99 (0.90–1.08)
Geriatric medicine assessment in the year prior to death	1.37 (1.14–1.63)	0.86 (0.69–1.06)
Malignancy in the preceding 5 years	1.00 (0.81–1.24)	1.18 (0.93–1.49)
Age (per 10 years)	0.71 (0.68–0.75)	1.03 (0.96–1.09)
Bedbound status	0.76 (0.64–0.91)	0.60 (0.49–0.72)
Urban vs. rural nursing home location	5.78 (4.09–8.15)	0.77 (0.61–0.97)
Number of nursing home beds		
100	1.00 *Ref*	1.00 *Ref*
100–199	1.88 (1.21–2.92)	0.79 (0.52–1.18)
≥200	2.58 (1.33–4.98)	0.70 (0.38–1.29)
Palliative care physician assessment in the year prior to death	0.45 (0.37–0.54)	0.72 (0.62–0.83)

[*] aOR: adjusted odds ratio. The multivariable logistic regression analysis modeled the exposure and each factor of interest adjusting for the exposure and all the remaining factors.

[†] Calculated for the 9,844 (36.1%) residents in the cohort who had a RAI-MDS 2.0 completed in the last 30 days of life

©2019 Stall NM et al. *JAMA Network Open*

**eAppendix 3.** Most Responsible Diagnoses for First Hospitalization in Last 30 Days of Life	
**Most responsible diagnosis**	**Number of episodes**
Z51: Other medical care	1,176
J69: Pneumonitis due to solids and liquids	950
A41: Other septicemia	602
J18: Pneumonia, organism unspecified	592
N39: Other disorders of urinary system	419
S72: Fracture of femur	286
J44: Other chronic obstructive pulmonary disease	177
E87: Other disorders of fluid, electrolyte and acid-base balance	148
I50: Heart failure	129
E86: Volume depletion	127
N17: Acute renal failure	127

©2019 Stall NM et al. *JAMA Network Open*
